# Loading Rate Has Little Influence on Tendon Fascicle Mechanics

**DOI:** 10.3389/fphys.2020.00255

**Published:** 2020-03-24

**Authors:** Michael V. Rosario, Thomas J. Roberts

**Affiliations:** ^1^Department of Ecology and Evolutionary Biology, Brown University, Providence, RI, United States; ^2^Department of Biology, West Chester University, West Chester, PA, United States

**Keywords:** viscoelasticity, tendon fascicle, rat tail, elastic energy (EE), resilience

## Abstract

Mechanically, tendons behave like springs and store energy by stretching in proportion to applied stress. This relationship is potentially modified by the rate at which stress is applied, a phenomenon known as viscosity. Viscoelasticity, the combined effects of elasticity and viscosity, can affect maximum strain, the amount of stored energy, and the proportion of energy recovered (resilience). Previous studies of tendons have investigated the functional effects of viscoelasticity, but not at the intermediate durations of loading that are known to occur in fast locomotor events. In this study, we isolated tendon fascicles from rat tails and performed force-controlled tensile tests at rates between ∼10 MPa s^–1^ to ∼80 MPa s^–1^. At high rates of applied stress, we found that tendon fascicles strained less, stored less energy, and were more resilient than at low rates of stress (*p* = 0.007, *p* = 0.040, and *p* = 0.004, respectively). The measured changes, however, were very small across the range of strain rates studied. For example, the average strain for the slowest loading rate was 0.637% while it was 0.614% for the fastest loading. We conclude that although there is a measurable effect of loading rate on tendon mechanics, the effect is small and can be largely ignored in the context of muscle-actuated locomotion, with the possible exception of extreme muscle-tendon morphologies.

## Introduction

**FIGURE 1:**
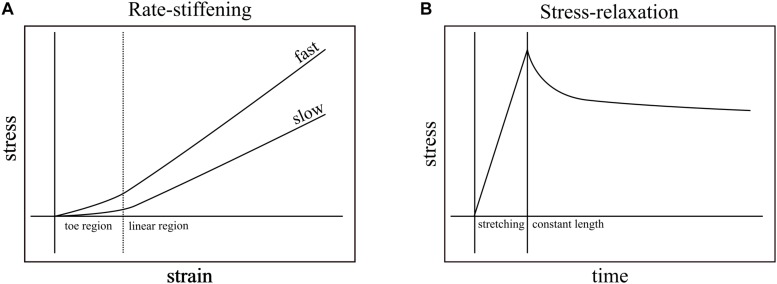
Rate-stiffening and stress-relaxation are two mechanical behaviors that result from the viscoelasticity of biological structures. **(A)** In rate-stiffening, faster rates of strain increase the effective stiffness of the structure, resulting in higher stress for a given strain. These effects can be seen in both the toe region (left of dotted line) and the linear region (right of dotted line). **(B)** Stress-relaxation results from holding a structure at constant length (right of solid line) upon the completion of stretching. Over time, the stress required to maintain constant length is reduced as the energy stored in the structure dissipates into the environment.

Nearly all biological structures, including those that comprise the musculoskeletal system, are viscoelastic – capable of deforming in response to load and reforming in its absence (elasticity) while exhibiting some sensitivity to load-rate (viscosity). For example, applying tensile strain at a constant rate to ligaments of rabbits (Crisco et al., 2002), monkeys (Noyes et al., 1974), and humans (Pioletti et al., 1998; Funk et al., 2000; Bonifasi-Lista et al., 2005) generally results in typical stress-strain curves: a relatively low stress “toe region” followed by linear increase in stress in response to strain (Figure 1A). These relationships vary, however, with strain rate. Ligaments, in general, exhibit higher slopes of stress-strain when faster strain rates are applied (Figure 1A; Haut and Little, 1969; Noyes et al., 1974; Crisco et al., 2002) and, when held at constant length after stretching, increasingly relax and dissipate stored energy the longer they are held (Figure 1B; Bonifasi-Lista et al., 2005). In addition to ligaments, rate-dependence has important mechanical consequences in musculoskeletal structures such as bones (Black and Korostoff, 1973; Lakes et al., 1979; Rimnac et al., 1993; Bowman et al., 1994). Because the ratio of viscous to elastic behavior varies with rate, the rate of loading has the potential to affect mechanical output.

Given that musculoskeletal structures are viscoelastic, tendons that are involved in multiple locomotor functions may modulate their mechanics in response to loading rate. In other words, if tendons are sensitive to loading rate, despite being completely passive structures, they may support different functions depending on the circumstances in which they are loaded. Hypothetically, this may manifest in a single tendon that can paradoxically enhance both energy storage and energy dissipation depending on whether force is provided by a slow contraction from the muscle or a fast impact from the ground.

Studies of the mechanical behavior of tendon have established viscoelastic behavior. The most common measurement of viscoelasticity involves stress-relaxation studies, which show declines in force in tendons that are stretched and then held at constant lengths (Figure 1B). These studies focus on the dissipative nature of tendons after loading (Tanaka et al., 1999; Provenzano et al., 2002; Elliott et al., 2003; Bonifasi-Lista et al., 2005; Duenwald et al., 2009), and rarely vary loading rate within a single study. While these studies provide insight into the viscoelastic effects that dominate at relatively long time scales (e.g., minutes), they do not typically test the viscoelastic effects that dominate during transient loading events such as the high impact landings that load turkey tendons within ∼60 ms (Konow et al., 2012) and the stretching of tendons prior to power amplification within ∼250 ms (Wainwright and Bennett, 1992; Lappin et al., 2006; Van Wassenbergh et al., 2008). Additionally, while some studies measured the effects of viscoelasticity while varying loading rate in collagenous structures, the two studies that are closest to matching rates found in transient events are still either slower (Robinson et al., 2004; load to failure in ∼400 ms) or faster (Crisco et al., 2002; load to failure in ∼4.6 ms) than physiological durations of loading that do not cause injury.

We tested viscoelasticity in the relatively unspecialized fascicles of rat tail tendons. Whole tendons can exhibit complex geometry along their lengths, thereby making stress calculations problematic; but, tendon fascicle geometry is relatively simple and more amenable to material testing. Additionally, there is a large body of work on rat tail tendon fascicles to which we can compare our results (Rigby et al., 1959; Screen et al., 2003; Haraldsson et al., 2009; Bruneau et al., 2010; Legerlotz et al., 2010). By isolating tendon fascicles and replacing their connected muscles with a dual force muscle motor, we were able to test how muscle-tendon interactions are affected by variation in loading rates that occur during transient events. Although viscoelasticity is traditionally measured by controlling strain, variation in tendon strain in a muscle-tendon unit is controlled by the muscle, a contractile element that acts as a force generator. Therefore, we programmed the dual mode motor to simulate muscle action by varying force directly, enabling fine control of both the amount and rate of force applied to the tendon fascicle at any given moment. We tested whether applying tensile stress at varying rates resulted in significant changes in three mechanical behaviors of the tendon fascicles: maximum strain, energy storage, and resilience.

## Materials And Methods

The tails of seven adult rats, which were euthanized for reasons unrelated to this study, were isolated for dissection and immediately submerged in Mammalian Ringer’s solution (115.00 mM NaCl, 4.70 mM KCl, 2.00 mM CaCl_2_, 1.20 mM MgSO_4_, 40.00 mM TRIS, 10.00 mM TRIS.HCl). Using a previously published method of dissection and extraction (Bruneau et al., 2010), from each tail, at least 20 tendon fascicles were dissected, wrapped in Ringer’s-soaked gauze, and kept frozen until tested.

**FIGURE 2:**
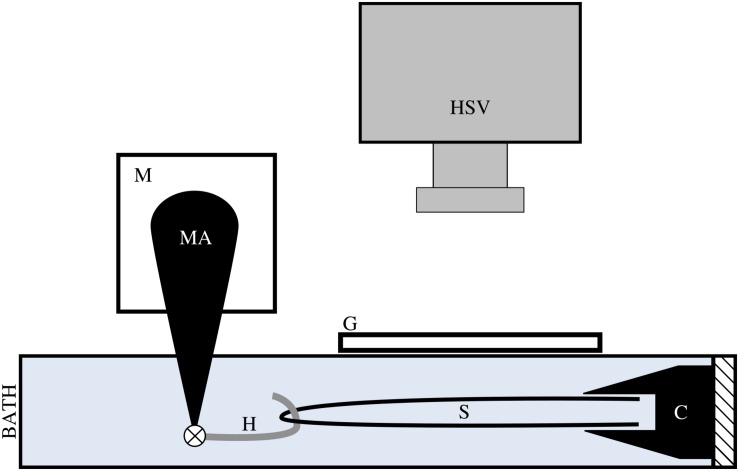
We applied tensile stress to a tendon fascicle sample (s) via a muscle motor (m) while recording video of the sample at 1000 Hz with a high speed video camera (HSV). We formed a loop with the tendon fascicle, gripped the two free ends in a clamp (c), and connected a hook (h) through the fascicle sample loop. The hook, which attached to the motor arm (MA) via a pin joint, was lined with plastic tubing to reduce friction on the sample during testing. During testing, the hook, fascicle, and clamp were fully submerged in mammalian Ringer’s solution. To prevent ripples at the surface of the solution, we placed a glass slide (g) directly above the fascicle sample.

Prior to testing, tendon fascicles were thawed at room temperature and immediately placed in mammalian Ringer’s solution for at least 10 min. Tendon fascicles were trimmed to a total length of ∼50 mm. We modified a previously published method of attaching the tendon fascicle to the testing chamber (Rigby et al., 1959). While submerged in solution, a single tendon fascicle was manipulated at both ends via forceps to form a half-loop. Both free ends of the tendon fascicle were gripped by a flat-faced copper clamp lined with Emery paper (Figure 2). Fascicles were transferred to an acrylic testing chamber filled with Ringer’s solution. In the chamber, the miniature clamp was secured to the acrylic wall, and a plastic-lined sterling silver hook was passed through the open loop of the fascicle. This hook was directly connected to the arm of a dual mode muscle level (305C-LR; Aurora Scientific; Ontario, Canada) via a pin joint. To ensure the tendon fascicle was not over-stretched during manipulation, we applied a preload of ∼0.050 N to the tendon fascicle and programmed the muscle motor to not exceed this force for the duration of preparation. Directly above the tendon fascicle, we placed a glass slide and added additional Ringer’s solution to the chamber until the liquid touched the bottom of the glass slide to prevent surface ripples during mechanical testing.

We calculated stress as the load divided by cross-sectional area at preload; therefore, an accurate estimate of cross-sectional area was required. We estimated the average cross-sectional area using fascicle width measured from a pair of orthogonal images. The testing chamber included a mirror that formed a 45-degree angle with respect to the horizontal surface of the testing chamber. We included two independent reference objects in the chamber for length calibration, one for each view. High speed video was captured from above via camera (Fastcam-X 1280PCI; Photron, IN, United States) mounted to a microscope (GZ6E; Leica, IL, United States).

Fascicle cross-sectional area was modeled as an ellipse using the following equation:

(1) A=π⋅a⋅b

where *a* and *b* are equal to half the width of the fascicle in each of the orthogonal views. To estimate the width of the fascicle, each image was manually preprocessed and were imported into R (R 3.3.1, Vienna, Austria) where estimates of *a* and *b* were calculated.

Previous studies have shown that strain calculated using motor distance can overestimate within-tendon strain by as much as 6% due to slipping at clamped surfaces (Haraldsson et al., 2005). Therefore, instead of relying on motor displacement for strain calculations, we measured strain using a non-contact, optical method. In preparation for visual strain measurement, we marked the tendon fascicle with multiple ink dots spaced roughly 5 mm apart. Because the tendon fascicle was looped, it formed two separate “strands” on which we placed dots. We offset the dots on each strand to provide an immediate visual indicator of slipping and failure during tests.

**FIGURE 3:**
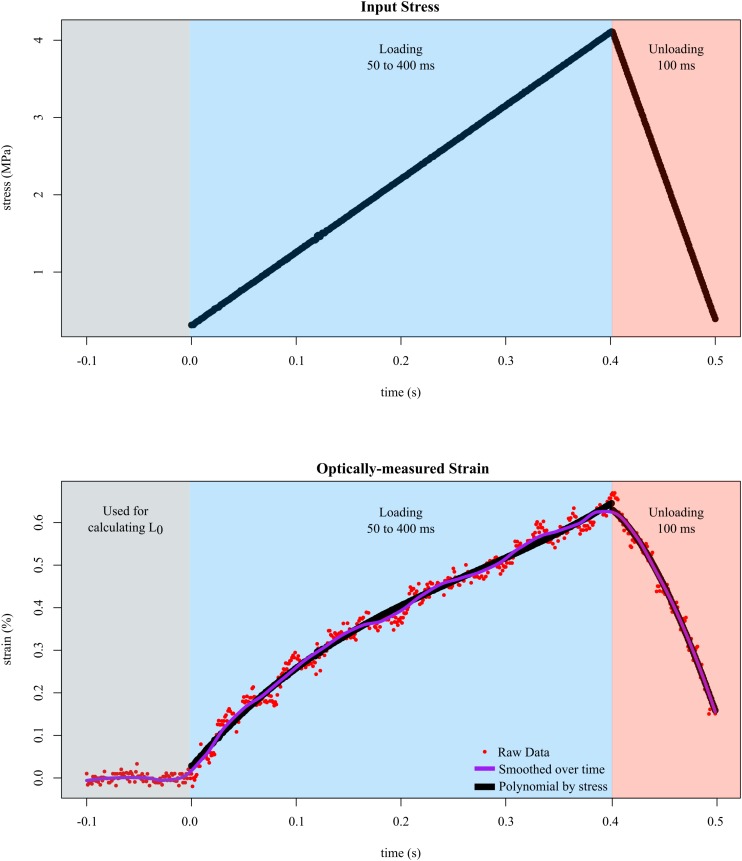
Tendon fascicles were linearly loaded to ∼4 MPa of stress at different rates while strain was optically-measured. To determine the mechanical response to stress rate, the duration of loading **(blue background)** was experimentally varied between 50 and 400 ms. After loading, the tendon fascicle was unloaded to its original stress within 100 ms in preparation for the next trial **(red background)**. Calculating strain required dividing length by L_0_, which was defined as the average distance between the markers over 100 ms prior to input stress **(gray background)**. The raw strain data **(red dots)**, were then smoothed via LOESS over time. The final phase of smoothing involved fitting 3rd order polynomials **(black lines)** to the stress-strain data of the loading data.

In all trials, we input different levels of stress while measuring the resulting strain. Tendon fascicles were preloaded to ∼0.050 N, which did not result in any visible changes in the crimping of the fascicles. We then inputted a triangle wave to control force such that a maximum of 4 MPa of stress was applied to the tendon fascicle. We chose a maximum stress of 4 MPa because previous studies have reported that under this stress, tendon fascicles remained linear, and plastic deformation was unlikely when tested repeatedly (Haraldsson et al., 2005). The duration of the rising portion of the triangle wave was experimentally varied from 50 to 400 ms at intervals of 50 ms to capture the natural range of variation in tendon loading. The duration of the falling portion of the triangle wave was fixed at 100 ms for all trials. Preliminary results showed no evidence of history effects; therefore, before each replicate, the order of these durations was randomized. Between each sequence of loading-unloading, the tendon fascicle rested in solution for at least 5 min, which provided enough time for the fascicle to recover from stress-relaxation effects. We collected data until either the tendon fascicle broke (replicates in which plastic deformation was obvious were removed prior to analysis) or at least three replicates of tensile stretch were collected. During each trial, we recorded high speed video at 1000 fps, which was synchronized with the muscle motor. In order to capture the resting length of the tendon fascicle, the high speed camera began recording 100 frames (100 ms) prior to each test (Figure 3).

Using a custom point tracker developed in R, we converted the synchronized high speed camera into a visual tensometer. The marks that were farthest apart on a single fascicle strand were selected manually by identifying a single pixel in each point. Because the boundaries formed by the ink mark were sensitive to optic parameters (lighting, lens effects, discretization of pixels etc.), we tracked the centroid of each marker, which was calculated as the mean x and y location of each pixel in each marker. This had two advantages. First, it minimized errors that resulted from inconsistent boundary identification. Second, because ink marks spanned multiple pixels in each image, calculating the centroid of a body of pixels allowed sub-pixel resolution of marker position.

After the centroids of both points were tracked, we calculated the distance, in pixels, between the centroids of each frame using the following equation:

(2) $$d\,i\,s\,t\,a\,n\,c\,e=\sqrt{{\left(c_{1}^{x}-c_{2}^{x}\right)}^{2}+{\left(c_{1}^{y}-c_{2}^{y}\right)}^{2}}$$

where $c_{1}^{x}$ and $c_{2}^{x}$ are the x values of the centroid of points 1 and 2, respectively, and $c_{1}^{y}$ and $c_{2}^{y}$ are the y values of the centroid of points 1 and 2, respectively. Finally, the distance between each marker was converted to strain by dividing each distance by the average distance between the markers recorded 100 ms prior to the start of each test.

The data were filtered in two ways. First, with respect to time, the position of each marker used to calculate strain was smoothed using Local Polynomial Regression Fitting (loess function in R) using an arbitrary smoothing parameter of 0.2. These data were then combined with stress, which was measured from the muscle motor. The dataset was then subdivided into the loading and unloading phases. Second, to each subset, 3rd order polynomials were fit to the stress-strain data. After polynomials were fit to the loading data, the maximum strain and stress of the loading polynomial was recorded for further analysis, and the unloading polynomial was forced to pass through this point, thereby connecting the loading and unloading curves at the transition point. The curves were then numerically integrated using the trapezoidal rule with a resolution of 0.00001 strain. To calculate resilience, we divided work loading by work unloading. There was evidence of slipping during the unloading of one fascicle; for those data, only maximum strain and energy stored were analyzed. We also estimated the elastic modulus of the tendon fascicle by dividing the total change in stress by the total change in strain (*E*_*secant*_). This value provides an average modulus of elasticity over the entire trial. Finally, to obtain the stress rate of loading, we divided the change in stress in the loading subset by ramp duration.

The data, which contain different numbers of replicates for each sample, were fit with a linear mixed-effects model in R using the nlme package (nlme version 3.1-128). To investigate the effect of stress rate on maximum strain we defined the fixed effect as *strain* ∼ *stress rate*. Due to the hierarchical nature of our dataset (n_observations = 175, n_treatments = 8, n_rats = 7), we defined replicate and rat ID as our two random effects. It is important to note that in our model, we define replicate as nested in rat ID (i.e., replicate 1 from one rat had no association with replicate 1 from another rat). We performed similar tests using identical random effects to determine the fixed effect of *energy storage* ∼ *stress rate* as well as *resilience* ∼ *stress rate*.

## Results

The average resulting strain across all trials was 0.57%. Given that all tendon fascicles were loaded to 4 MPa of stress, the average Young’s Modulus of Elasticity (*E*_*secant*_) of all trials was 691 ± 148 MPa (Table 1). The range of strain experienced by all fascicles was 0.458–0.874%.

**TABLE 1:** The resulting strain rates from our testing protocol are greater than those used by other studies of rat tail tendon fascicles; but, our average measures of Elastic Modulus are comparable to previously reported data.

Study	Strain rate (%/s)	Modulus ± SD (MPa)
Current study	2.18–9.16	691 ± 148
Legerlotz et al., 2010	1	1000 ± 165
Screen et al., 2004	0.33	NA
Rigby et al., 1959	0.016–0.33	NA
Haraldsson et al., 2009	∼0.12	641 ± 30

**FIGURE 4:**
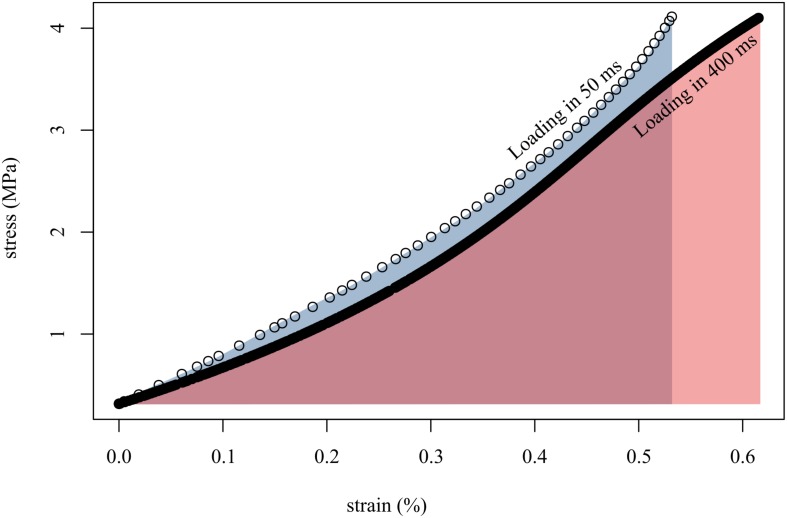
Loading tendon fascicles quickly **(in 50 ms; open circles)** resulted in higher stiffness **(slope of open circles)** and lower final strain than when loading the same fascicles slowly **(in 400 ms; solid black line)**. This translated to more work done on the fascicle when loaded slowly **(red area)** than when loaded quickly **(gray area)**. Shown here are representative data from a single tendon fascicle.

**FIGURE 5:**
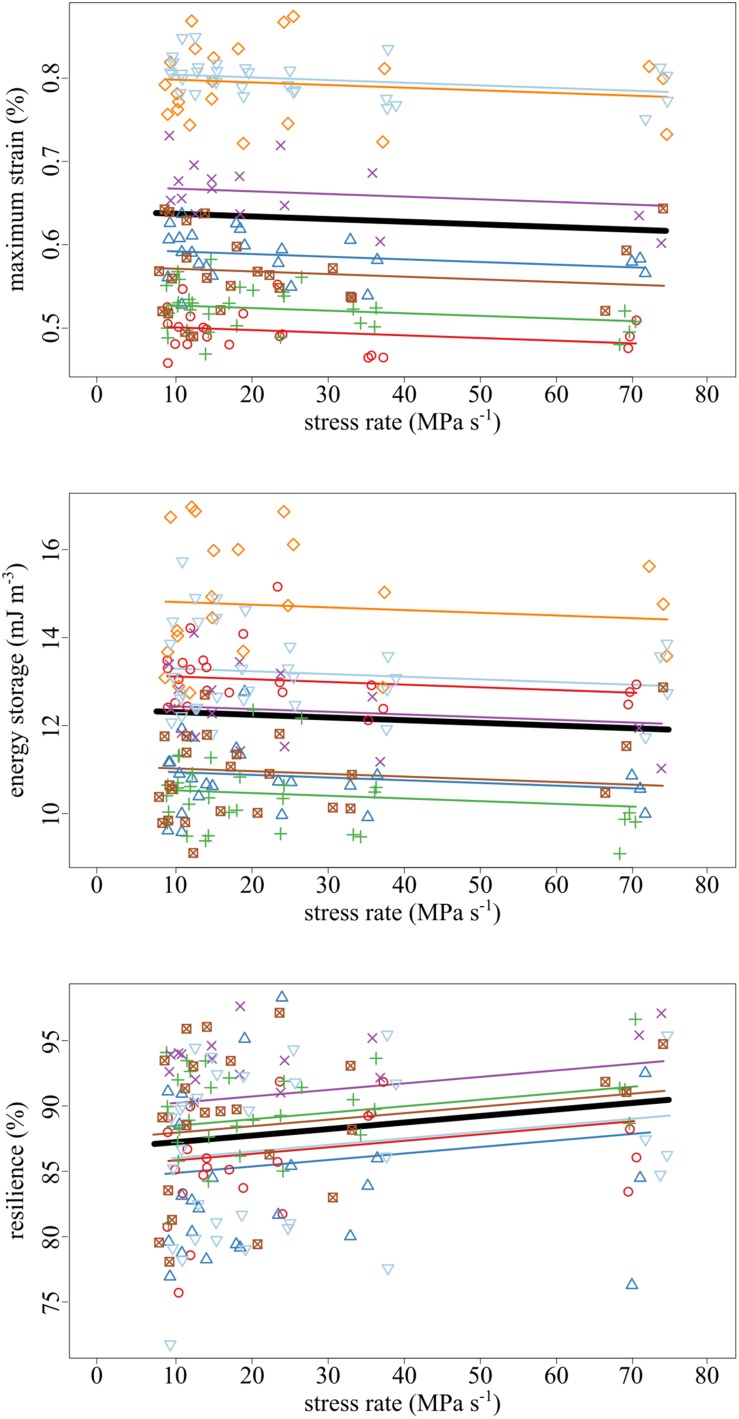
As the stress rate of loading increased, both the maximum strain and strain energy storage decreased while the resilience increased. Data collected from the same tendon fascicle are represented by unique colors and symbols. A linear mixed-effects model was fit to the data to account for the hierarchy due to non-independence within replicates and samples. The thick black lines represent significant fixed effects of maximum strain (*p* = 0.007), energy storage (*p* = 0.040), and resilience (*p* = 0.004) regressed against stress rate. Thin lines represent within- individual relationships and are colored identically to the raw data to which they correspond.

The amount of maximum strain and stored energy in the tendon fascicles was negatively correlated with stress rate (Figures 4, 5). These effects are demonstrated by the significantly negative relationships of strain (slope = −3.19 × 10^–2^, *SE* = 1.16 × 10^–2^, *p* = 0.007) and energy storage (slope = −6.13 × 10^–3^, *SE* = 2.95 × 10^–3^, *p* = 0.040) to stress rate (Figure 5). Compared to the slowest stress rates, at the fastest stress rates, maximum strain decreased from 0.637 to 0.614%. This was accompanied by a 3.48% decrease in energy storage. Conversely, increasing stress rate increased resilience (slope = + 4.98 × 10^–4^, SE = 1.69 × 10^–4^, *p* = 0.004).

## Discussion

The goal of this study was to determine whether rate of loading significantly influenced the mechanical behavior of tendon across a physiological range of loading. Our choice of loading rates were meant to span from some of the fastest loading rates that have been observed in vertebrates during movement to a relatively slow loading rate meant to represent the kind of loading that may occur in “ordinary” movements. We found that when tendon fascicles were loaded at faster rates, they stretched less, stored less energy, but were more resilient. These data suggest that loading rate does have the potential to influence tendon mechanical behavior *in vivo*, and thus muscle function via muscle-tendon mechanical interactions. The magnitude of the effect of loading rate was small, but, depending on factors such as the ratio of tendon length to muscle length in the muscle-tendon unit (MTU), small viscoelastic effects of tendon can affect muscle mechanical function.

### Potential Benefits of the Rate-Sensitivity of Tendons

When loaded at fast rates, maximum strain and stored energy were reduced relative to slower stretches. Either of these has the potential to contribute to failure. Given that tendons are most susceptible to sports-related injury during high acceleration/deceleration exercise (Soldatis et al., 1997), the reduction of strain and stored energy at high loading rates has the potential to aid in reducing the chance of tendon failure. Studies of primate anterior cruciate ligaments that found that, at high rates of loading, ligaments failed at higher loads and greater elongations, suggesting some protective mechanism associated with high rates of loading (Noyes et al., 1974). At slow rates of loading, we found that maximum strain and energy storage increased. Given that the tendons that are used for elastic energy storage are loaded relatively slowly (Wainwright and Bennett, 1992; Lappin et al., 2006; Van Wassenbergh et al., 2008), the mechanical response of tendon fascicles complements their function at slow loading rates, allowing the tendon fascicles to stretch more and store more energy for a given amount of input stress. Tendon fascicles potentially limit stored energy when it is harmful and increase stored energy when it is of use; but, to determine the ability of tendons to modulate mechanical response, it is important to consider the size of these effects and their ability to influence organismal-level mechanics.

### Estimating the Effects of Tendon Fascicle Viscoelasticity on MTU Mechanics

When a given amount of force is applied to a biological material (such as when a muscle stretches a tendon in a fixed-end contraction), lower stiffness results in higher strains. In the MTU, this results in comparatively more tendon stretch as stress rates decrease. In the context of muscle force generation, the sensitivity of tendon stretch to stress rate has the potential to affect muscle dynamics. For example, in a fixed-end contraction, a tendon that stretches to longer lengths would necessitate more muscle fiber shortening. Changes to muscle fiber shortening strain or speed can influence muscle force output via the force-length and force-velocity behaviors of muscle. We found that tendon fascicle strain decreased 0.023% from 0.637 to 0.614% strain between our slowest and fastest experimental stress rates. On its own, this ∼4% change in tendon strain would seem to suggest a minor effect of loading rate on muscle fiber dynamics. However, depending on the dimensions of the muscle and tendon, the effect has the potential to be significant.

The influence of variation in tendon strain on muscle fiber strain will depend on the ratio of muscle fiber and tendon lengths. If we take as an example the human gastrocnemius we can calculate the maximum possible effect of loading rate that our data would suggest. Resting length values of 225 and 60 mm have been reported for the gastrocnemius tendon and fiber length (medial gastrocnemius) respectively (Maganaris and Paul, 2002; Lichtwark and Wilson, 2006), for a tendon/fiber length ratio of 3.6. Achilles tendon strains during running and maximum voluntary contractions have been reported as ∼4% (Maganaris and Paul, 2002; Lichtwark and Wilson, 2006). Our maximum expected change in strain from slow to fast loading of 4% would mean that loading rate has the potential to change achilles tendon strain *in vivo* by 0.04 × 0.04 = 0.0016, or 0.16%. Given the tendon/muscle fiber length ratio, this could alter fiber strain by 0.16 × 3.6 = 0.58%. Thus, loading rate under these conditions for this muscle seems unlikely to have a meaningful effect on muscle fiber dynamics.

Tendon to fiber length ratios vary among muscles, and for extreme systems the very small effects of loading rate observed here have the potential to significantly influence muscle fiber strain during contraction. In the extreme case of the flexor digitorum superficialis muscle in horses, which has been estimated to operate with a tendon that is 100 times longer than the resting length of the muscle (Biewener, 1998). The same calculation used above would yield, for this muscle, a 16% difference in muscle fiber strain when the tendon is loaded under slow vs. fast loading. Such a change would have significant influence on muscle fiber mechanical output via force-velocity and force-length behavior.

It seems unlikely that the measured significant increase in resilience in response to stress rate has an important impact on the mechanics of the MTU *in vivo*. At the slowest stress rates (where resilience is lowest), resilience remained relatively high at 87.23%, demonstrating that most elastic energy stored in the tendon fascicle was returned with little dissipation. Our least resilient tests fall slightly below the reported values for collagen found in mammalian tendon (90%; Pollock and Shadwick, 1994), for turkey hind limb tendons (90–94%; Matson et al., 2012), and for elastin fibers from bovine ligaments (90%; Aaron and Gosline, 1981). For comparison, cyclical strain tests that were conducted on the nuchal ligament of cows showed a decrease in resilience from 76 to 31% when increasing the frequency from 1 to 31 Hz (S. A. Wainwright et al., 1965). Our choice of unloading duration may have influenced our results to some extent (longer durations of unloading could lead to lower values of resilience); but, given that the duration of elastic recoil is 20–50 ms in jumping anurans (Astley and Roberts, 2014) and on the order of 20 ms in the tongue projection of chameleons (de Groot and van Leeuwen, 2004), our unloading duration of 100 ms provides conservative estimates. The relatively high values for resilience of rat tail tendon fascicles across all our experimental stress rates indicated that viscoelasticity did not play a major role in energy dissipation. Additionally, it is important to note that resilience in the tendon fascicles is lowest at slow rates of loading; therefore, even though our results suggest that more energy is stored in tendon fascicles with slow stress rates, less of that energy is recovered during unloading, potentially counteracting the energy storage benefits of stretching tendon slowly.

### Limitations

Because tendons are hierarchical structures, it is possible for viscoelastic effects to emerge at various levels of organization; therefore, it is important to consider the benefits and limitations of investigating viscoelasticity solely at the fascicle level. A benefit of tendon fascicles studies is the abundance of rat tail tendon fascicle studies, thereby providing measurements that are directly comparable to our own (Hansen et al., 2002; Haraldsson et al., 2009; Legerlotz et al., 2010). For example, Elastic Moduli obtained in this study were consistent with previously published values. In all tests and across all rates of loading, the average value for *E*_*secant*_ was 691.18 ± 148.88 MPa, which was consistent with previously published calculations for Young’s modulus of elasticity (Table 1).

Although not perfectly elliptical, the cross-sectional area of a tendon fascicle in rat tail tendons is generally simpler and more uniform in shape than that of whole tendon. The simplified morphology of tendon fascicles reduces potential errors in the calculation of stress. The major drawback of focusing on tendon fascicles, however, is that our results may not necessarily reflect mechanics of the whole tendon. For example, the viscoelastic behavior of horse tendons has been attributed to the interfascicular matrix (Thorpe et al., 2015, 2016, 2012). Additionally, the viscoelastic effects at play during stress-relaxation of rat tail tendons are highly dependent on the effects of fibers sliding past each other (Screen, 2008; Gupta et al., 2010). These effects are completely ignored when testing solely at the fascicle level. Although some studies have shown that the fascicles of some tendons likely function as independent structures with little to no interaction occurring between fascicles (Haraldsson et al., 2008), it is important to remember that any mechanical interaction between tendon fascicles is not captured in the present study.

The applied stresses and measured strains in this study were much lower than those typical of most studies of whole tendons. Because our mechanical tests required repeatable trials per sample and multiple replicates without failure, we used conservative upper bounds for maximum stress. Previous studies have found that applying up to 4 MPa of stress during preconditioning did not alter rat tail tendon fascicle mechanics in subsequent trials (Haraldsson et al., 2005); therefore, we chose to apply no more than 4 MPa of stress during all of our trials. As a result, the maximum strain achieved across all trials was less than 1% Additionally, in our preliminary tests, we were not able to exceed 1.5% strain without failure. Although previous studies have reported up to 4% (Rigby et al., 1959) strain and 12 MPa of stress before indication of failure of tendon fascicles (Haraldsson et al., 2009), we were not able to replicate these results. It is likely that our method of testing caused concentrations of stress and/or strain in the tendon fascicle near the clamp, resulting in failure at much lower strains and stresses than previously reported. Despite the possible stress concentrations in our samples, when stress did not exceed 4 MPa, our data showed no indication of plastic deformation or failure.

It is also important to note that because we restricted our tests to low stress values, we could not directly measure the effects of viscoelasticity at the normal operating levels of strain. In reality, high rates of stress lead to substantial tendon strain within short durations, however, our data are only representative of the “toe region” of rat tail tendon stress-strain curves, which occurs within 1–2% strain of the whole tendon (Hansen et al., 2002; Screen et al., 2004). Despite our maximum strain falling slightly below this range, most reported estimates of strain (including the measurements of the “toe region” mentioned above) are made using grip-to-grip distance whereas our visual method measured strain mid-substance. Studies have shown that strain measured via grip-to-grip distances is consistently higher than strain measured mid-substance (Haraldsson et al., 2005). Indeed, the values of maximum strain we measured using our optical method was on average 0.278% less than the strain measured using grip-to-grip distances (see Supplementary Figure S1). A major design choice in this study was to avoid failure by applying small stresses to the tendon fascicles; however, given that previous work on mice tail tendon fascicles showed small rate effects on elastic modulus but large sensitivity to yield stress (Robinson et al., 2004), future work should test viscoelasticity at higher stresses and strains.

## Conclusion

Despite the significant effects of stress rate on maximum strain, energy storage, and resilience, these effects were not large enough to dominate non-specialized muscle-tendon mechanics at rates relevant to transient loading events. These data complement previous studies that demonstrated long-range rate dependence via tensile creep and stress-relaxation tests in collagenous tissues. In these cases, the effect of rate has large effects on the mechanical properties of these tissues. It is likely then that the importance of viscoelastic effects gradually increases with the duration of loading. At the durations of tendon loading that are likely to occur during landing and power amplification, however, the present results suggest that viscoelasticity can be largely ignored.

## Data Availability Statement

The datasets generated for this study are available on request to the corresponding author.

## Ethics Statement

Samples were collected from animals euthanized for unrelated studies. All animal use for these unrelated studies was approved by the Brown University Institutional Animal Care and Use Committee.

## Author Contributions

MR designed the experiment and collected and analyzed the data. TR helped design the experimental rig and provided guidance. MR and TR both contributed to the writing and editing of the manuscript.

## Conflict of Interest

The authors declare that the research was conducted in the absence of any commercial or financial relationships that could be construed as a potential conflict of interest.
