# Supplementary material for: Loading Rate Has Little Influence on Tendon Fascicle Mechanics
Source: Front Physiol. 2020 Mar 24;11:255. doi: 10.3389/fphys.2020.00255 (PMC7105874; doi:10.3389/fphys.2020.00255)
Supplement: Supplementary file 1 [file Data_Sheet_1.PDF]

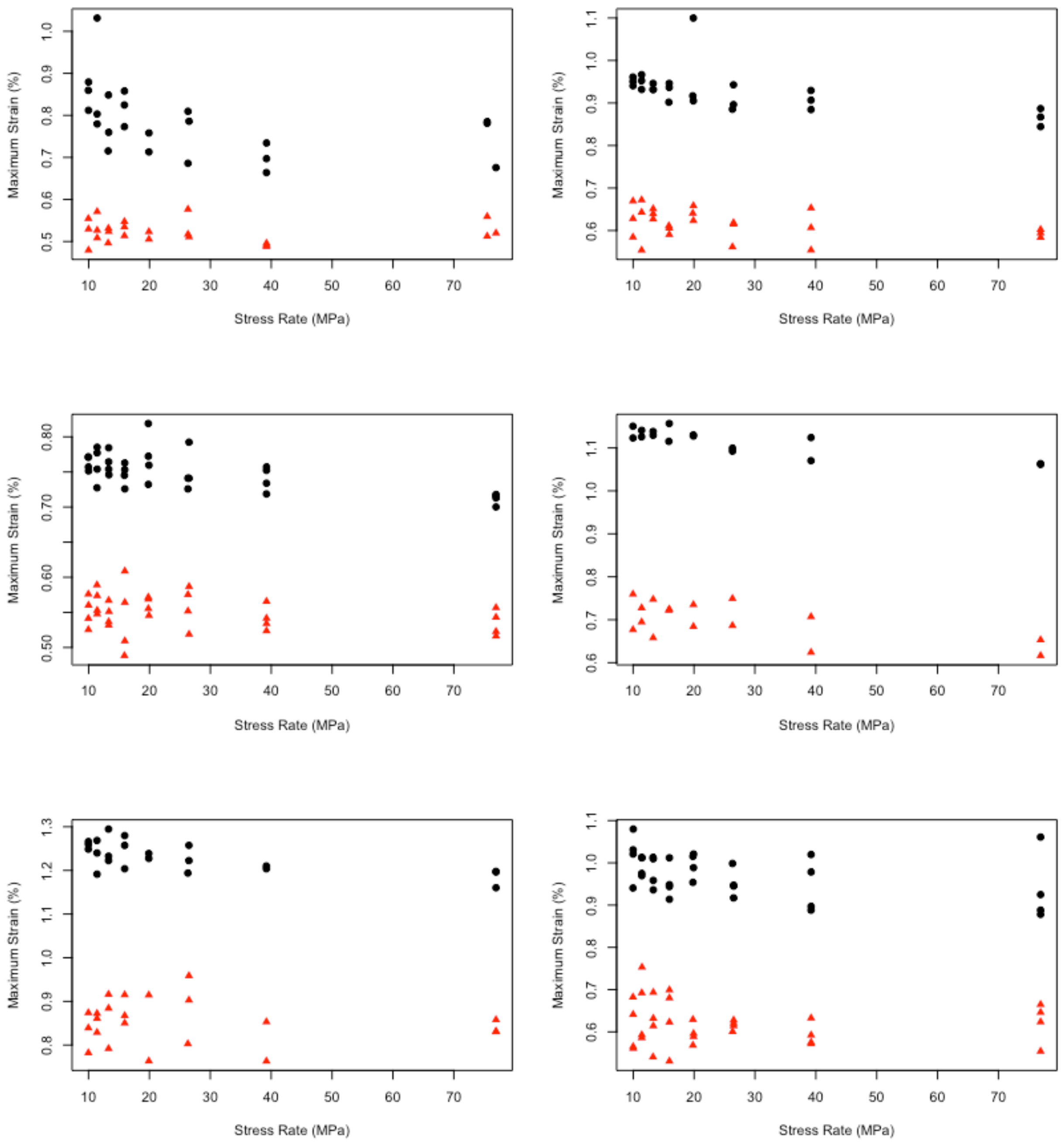

Figure S1. The maximum strain of our experiments was higher when measured using grip-to-grip distances (black circles) than when measured optically (red triangles). Each panel represents data collected from a single rat tail tendon fascicle. On average, grip-to-grip measures of strain were 0.278% higher than optical measures.
